# Gain or loss? Examining the dual effects of small talk on employees’ safety performance

**DOI:** 10.3389/fpsyg.2025.1622001

**Published:** 2025-08-13

**Authors:** Pan Liu, Mingyu Zhang, Ru Guo, Caili Duan, Bixiang Shi, Bolu Wei

**Affiliations:** ^1^China University of Geosciences (Beijing), Beijing, China; ^2^Beijing Jiaotong University, Beijing, China; ^3^Open University of China, Beijing, China; ^4^Hebei Normal University, Shijiazhuang, China; ^5^Center for Strategic Studies, Chinese Academy of Engineering, Beijing, China; ^6^China Academy of Safety Science and Technology, Beijing, China

**Keywords:** small talk, safety performance, COR theory, psychological availability, work absorption

## Abstract

**Introduction:**

Small talk, an informal social interaction in workplaces, has been overlooked in research on safety performance, which traditionally focuses on organizational factors (e.g., safety climate, leadership). Grounded in Conservation of Resources (COR) theory, this study explores its dual effects on employees’ safety performance, proposing that small talk may enhance safety performance via resource gain (psychological availability) and undermine it via resource loss (work absorption).

**Methods:**

Data was collected from 136 full-time employees in China through self-reported questionnaires, measuring small talk, psychological availability, work absorption, and safety performance. Path analysis and bootstrapping approach were conducted to test for the direct and indirect effects of small talk.

**Results:**

The results confirmed the dual effects of small talk on safety performance: small talk positively predicted safety performance through increasing psychological availability, while negatively predicted safety performance by reducing work absorption.

**Discussion:**

This study advances safety research by highlighting small talk as a micro-level determinant of safety performance, and enriches COR theory by illustrating resource gain/loss mechanisms in social interactions. Practically, it offers insights for managing informal communication to balance relational benefits and task focus, optimizing workplace safety.

## Introduction

Safety performance is a critical metric for evaluating the effectiveness of individuals and organizations in maintaining workplace safety (Fernández-Muñiz et al., 2017). At the individual level, it encompasses a multidimensional framework of behaviors that proactively safeguard oneself, colleagues, and the work environment. Griffin and Neal (2000) conceptualize this framework through two distinct dimensions: safety compliance and safety participation. Safety compliance involves adherence to essential safety protocols, such as following standardized operating procedures and consistently using personal protective equipment (Neal and Griffin, 2006). Safety participation, conversely, refers to voluntary actions that cultivate a collective safety culture, including assisting coworkers with safety challenges, attending safety training workshops, and advocating for safety improvements in team meetings (Wei et al., 2016).

Empirical studies consistently demonstrate that both dimensions significantly reduce workplace accidents and injuries, underscoring their dual importance in fostering safer work environments. To enhance employees’ safety performance, scholars have long sought to identify determinants of safety performance, focusing primarily on organizational factors (e.g., perceived safety obligations, Mullen et al., 2017; safety climate, Kalteh et al., 2021), leadership (e.g., safety leadership, Wu et al., 2011), and individual-level factors (e.g., cognitive ability, Guo et al., 2019; emotional intelligence, Lu and Kuo, 2016). These studies have advanced our understanding of structured influences on safety but have overlooked the roles of informal social interactions (e.g., small talk or chit-chat) in workplace safety.

Small talk, defined as superficial or trivial communication that does not involve task-related exchange of information, is ubiquitous in workplace safety (Methot et al., 2021). Despite its seemingly trivial nature, this type of social interaction can have a profound impact on employees’ safety performance. On the one hand, small talk facilitates amiable interactions and assists employees transition between roles and activities, thereby creating a sense of belonging and connection (Coupland et al., 1992). And, small talk is beneficial for the enhancement of positive social emotions such as friendly feelings, respect, sympathy, and pride (Kitayama et al., 2000). These positive emotions contribute to resources acquisition, allowing employees to concentrate on safety behaviors (Sonnentag, 2001; Wei and Mao, 2023). On the other hand, small talk can interrupt employees’ work, impeding or delaying their progress on work tasks and reducing their cognitive work engagement (Jett and George, 2003). This is because small talk involves the mutual awareness and participation of both parties, which momentarily diverts employees from tasks (Goffman, 1967). The scripted and routinized nature of small talk facilitates role exit, causing employees to ‘go on autopilot’ and cognitively detach from their work (Ashforth et al., 2001). Consequently, small talk can reduce the time and energy available for employees to engage in their safety behaviors, potentially affecting their performance (Jett and George, 2003). In this manner, small talk can exert a dual influence on employees’ safety performance.

**Figure 1:**
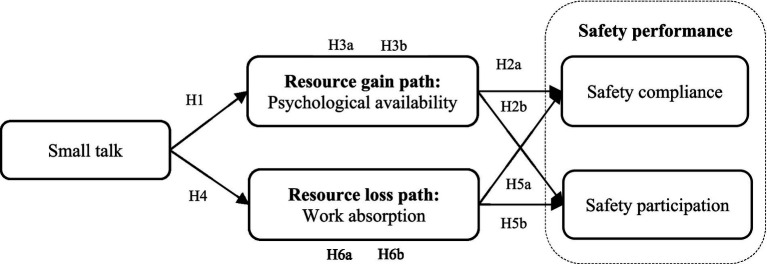
Conceptual model.

Conservation of resources theory illustrates that depletion or potential depletion of resources can impose stress on individuals, whereas the acquisition of resources can lead to positive psychological experiences (Hobfoll, 1989). The same situation can function as both a source of resource depletion and a source of resource acquisition (Hobfoll et al., 2018). Correspondingly, we postulate that small talk has a double-edged sword effect on employees’ safety performance. Using this argument, we further elaborate on the underlying mechanisms of the effects of small talk on individuals’ safety performance from two perspectives of resource loss and resource acquisition. The resources acquisition path implies that small talk is positively associated with employees’ safety performance through improving psychological availability, defined as an individual’s mental state and capacity to engage and focus on tasks. When employees engage in small talk, they can strengthen their sense of belonging, which can boost their overall job satisfaction (Kitayama et al., 2000; Methot et al., 2017). Moreover, small talk can promote the exchange of information, facilitating better collaboration and teamwork (Van Kleef, 2009). It also serves as a way to break the ice and ease tensions, making interactions more agreeable and productive, thereby augmenting psychological availability (Ferris et al., 2008). Hence, psychological availability can trigger a motivational process and, subsequently, positively affect employees’ safety performance. The resource loss path indicates that small talk is negatively related to employees’ safety performance by decreasing work absorption, defined as a state of fully concentration and deep immersion in work. As an extra-role behavior, small talk consumes psychological and cognitive resources, thereby interrupting work and distracting employees from tasks, which can lead to a reduction in safety performance (Kahn, 1990; Jett and George, 2003). Above all, our proposed model is illustrated in Figure 1.

To advance the research, we investigated both the negative and positive indirect effects of small talk on employees’ safety performance, including work absorption and psychological availability. This study proffers several contributions to the literature: First, this study departs from traditional research that focuses on leadership style, organizational culture, and job characteristics by emphasizing the role of individual behaviors, specifically small talk, in influencing safety performance. This shift in focus accentuates the vital significance of social interactions and communication in workplace safety, thereby complementing existing theories that may have hitherto overlooked these aspects (Aktas and Kagnicioglu, 2023; Yang et al., 2024). Second, this study challenges the long-held view of small talk as uniformly negative or unproductive by revealing its intricate nature. It illustrates that small talk can foster social cohesion and boost psychological resources of employees, yet simultaneously has the potential to disrupt work processes and undermine safety awareness (Denner et al., 2025; Methot et al., 2021). It provides a more comprehensive understanding of small talk in safety performance, advocating for a balanced consideration of its benefits and costs. Third, this study integrates the COR theory to explicate the mechanisms through which small talk affects safety performance. This theoretical prism offers a nuanced explanation of how small talk can influence employees’ safety performance, thus deepening our understanding of the nexus between social interactions and workplace safety.

## Theoretical background and hypotheses

### Conservation of resources theory

Small talk has dual effects on safety performance, and the COR theory furnishes a theoretically integrated framework, detailing the circumstances under which small talk either impairs or benefits employees’ safety performance. The COR theory postulates that employees in possession of ample resources have greater opportunities to obtain additional resources and derive benefits therefrom, whereas those bereft of crucial resources are more liable to endure subsequent losses and perceive threats, such as stress (Hobfoll, 1989). In line with the COR theory, one of the basic human necessities is to acquire and accumulate resources for the purpose of conserving other important resources that are vital for attaining higher-level goals or an ideal future state (Hobfoll, 2011; Hobfoll et al., 2018). The COR theory further expounds that when individuals are confronted with threats to their essential resources, endure the loss of these resources, or fail to replenish their resources adequately, stress is prone to emerge. Notably, resource loss is more conspicuous than resource gain as it constitutes a significant hazard to survival and affects people more expeditiously (Ojo et al., 2021).

Consequently, the core hypotheses of dual paths are formulated based on COR theory, illustrating that two separate underlying processes are involved in small talk. On the one hand, we consider psychological availability and elaborate its mediating role in the resource acquisition path. Psychological availability reflects individuals’ physical, emotional, or psychological resource level (May et al., 2004). Positive social interaction in the workplace can exert beneficial effects on employees’ psychological state (i.e., psychological availability) and generate desirable outcomes (Collins, 2004; Heaphy and Dutton, 2008). Thus, in this study, our emphasis lies on enhancing the psychological state by means of small talk. On the other hand, we consider work absorption and elaborate its mediating role in the resource loss path. Small talk consumes time and energy that employees should have invested in their work, making it difficult for them to be absorbed in safety behaviors. That is, small talk distracts employees from safety work, thereby hindering safety performance (Rothbard, 2001). In sum, we are devoted to test whether small talk affects safety performance via psychological availability and work absorption.

### Resource acquisition path: the mediating role of psychological availability

Psychological availability refers to an individual’s mental state and ability to be engaged and focused in a task (May et al., 2004), which is of crucial significance in determining employees’ workplace performance. Grounded in COR theory, we assert that small talk can exert positive effects on employees’ psychological availability.

First, small talk can function as a form of social resource. In organizational settings, social interactions constitute a vital part of work life. Small talk presents employees with an opportunity to establish connections with others, foster relationships, and foster a sense of belonging (Kitayama et al., 2000). The social interaction can augment an individual’s psychological resources, since it provides emotional support and a sense of community (Collins, 2004; Heaphy and Dutton, 2008). When employees engage in small talk about non-work-related topics such as hobbies or weekend plans, they may feel more closely affiliated with their colleagues and experience an elevation in morale. The enhanced sense of belonging and emotional support can contribute to higher levels of psychological availability.

Second, small talk can serve as a stress-relieving mechanism. Safety work is demanding and stressful, and small talk offers a brief respite from the pressures of the job (Kim et al., 2018). By engaging in light-hearted conversation, employees can temporarily divert their attention from work-related stressors and unwind (Owens et al., 2016), which assists in replenishing psychological resources. A short chat about the latest movie or a humorous anecdote can lighten the mood and reduce stress, allowing employees to return to their work with a refreshed sense of energy and focus. Reducing stress can facilitate increased psychological availability, as employees are better equipped to cope with the demands of their safety work when they are less stressed.

Third, small talk can boost cognitive flexibility. When employees partake in diverse conversations during small talk, they are exposed to different perspectives and ideas (Elsbach and Hargadon, 2006). And, this exposure can stimulate cognitive processes and enhance creativity (Trougakos et al., 2008). A discussion about a current event or a new technology may inspire employees to think differently about their work tasks or problem-solving approaches. The increased cognitive flexibility is advantageous for higher levels of psychological availability, as employees are more capable of adapting to changing work demands and challenges. Hence, we propose:

*Hypothesis 1: small talk is positively related to psychological availability.*

Psychological availability reflects a state in which individuals can channel their psychological, intellectual, and emotional resources into job performance (Kahn, 1990), and it can assist them in tackling the requirements necessary for proactive behaviors. When individuals are psychologically available, they possess physical, emotional, or psychological resources and thus have enhanced energy to take initiatives (Liu et al., 2021). Consequently, we assumed that psychological availability is positively related to employees’ safety performance.

The COR theory emphasizes that employees with plentiful resources have more prospects of obtaining additional resources and reaping benefits (Hobfoll, 1989). Psychological availability enables individuals to allocate their cognitive, emotional, and physical resources more effectively towards safety-related activities. When employees are psychologically available, they are more inclined to focus their attention on safety tasks and procedures (Kahn, 1990). The increased focus enables them to identify potential hazards more rapidly and implement appropriate preventive measures. An employee who is psychologically available is more likely to spot a loose wire or a slippery floor and take immediate action to rectify the problem, thereby reducing the accident risk. Moreover, psychological availability bolsters an individual’s capacity to manage stress and cope with challenging situations (Kahn, 1990). In a work environment, stress frequently gives rise to distractions and errors, which can negatively affect safety performance (Oaten and Cheng, 2005; Binyamin and Carmeli, 2010). When employees are psychologically available, they are better equipped to regulate their emotions and manage stress, allowing them to maintain a clear mind and make rational decisions. Psychological availability can allow them to stay focused on safety and avoid making mistakes that could lead to accidents. Hence, we propose:

*Hypothesis 2: psychological is positively relate to employees’ safety compliance (2a) and safety participation (2b).*

We also expect that small talk will be positively associated with safety performance via psychological availability. Small talk, as a form of social interaction, can assist employees in accumulating and augmenting various resources (Methot et al., 2021). First, employees can foster rapport and a sense of belonging among coworkers through casual conversations about non-work-related topics. The emotional support functions as a resource that can boost psychological availability (Muraven and Baumeister, 2000; Trougakos et al., 2014). When employees feel supported and connected, they are more prone to be in a positive psychological state, which subsequently renders them more receptive to safety compliance and safety participation. Second, employees can take a break from intense work tasks and unwind for a short moment by engaging in small talk (Westman et al., 2004), which can help relieve stress and replenish psychological resources. When stress is reduced, employees are better able to concentrate on safety and perform their work in a calmer and composed manner, which indirectly improves safety performance. Hence, we propose:

*Hypothesis 3: small talk promotes employees’ safety compliance (3a) and safety participation (3b) via psychological availability.*

### Resource loss path: the mediating role of work absorption

Although small talk serves as a vital lubricant in social interaction, it can also disrupt employees’ work (Jett and George, 2003). Small talk typically consumes the time, energy, and cognitive resources that employees should have dedicated to their work, thereby making it challenging for them to be absorbed in safety-related tasks. In this regard, when employees are engrossed in small talk about gossip, weather, and other topics, they are unable to fully engage in the task at hand and completely indulge in work (Jett and George, 2003). That is, small talk inevitably impacts work absorption, which is characterized by being fully concentrated and deeply engrossed in work, whereby time passes quickly and employees have difficulties with detaching themselves from work (Schaufeli et al., 2002; Kahn, 1990; Rich et al., 2010). Employees with work absorption can fully unleash their potential and achieve efficient work output. However, small talk may disrupt this state and easily distract employees from safety-related work.

To some extent, when individuals possess sufficient psychological resources, such as time and energy, they usually exhibit higher work engagement. However, frequent interruptions can severely interfere with employees’ focus on their work roles, thus impacting their work efficiency and performance (Kahn, 1990). Given that small talk is essentially an interactive behavior involving mutual awareness and participation of both parties (Goffman, 1967), it inevitably causes employees to be temporarily detached from their current tasks. Moreover, small talk is scripted and routinized, thereby leading individuals to unconsciously deviate from their work (Ashforth, 2001; Ashforth et al., 2001; Ashforth and Fried, 1988). When employees decide to take a short break from safety-related work, they may choose to do so through small talk. However, considering that cognitive engagement requires continuous behavioral motivation and attention as support (Lin et al., 2013), small talk is likely to interrupt employees’ attention and thus affect their work absorption. Therefore, we propose:

*Hypothesis 4: small talk is negatively related to work absorption.*

Based on the COR theory, work absorption is of vital significance in promoting safety performance. Work absorption entails individuals fully concentrating on their tasks (Rothbard, 2001). Such intense focus and attention allow employees to better allocate their cognitive resources to safety-related activities. When fully engaged in the work, employees can swiftly identify and respond to potential safety hazards, thereby reducing the accidents probability and ensuring a safe working environment (Griffin et al., 2007). That is, work absorption enables employees to perform their tasks more efficiently and effectively, including carrying out safety procedures and protocols with greater precision and thoroughness. Thus, we propose:

*Hypothesis 5: work absorption is positively related to safety compliance (5a) and safety participation (5b).*

Small talk can impede employees’ safety performance. Work disruptions caused by small talk can shift employees’ attention to activities that are not beneficial for work they are currently performing. The interruptions can leave employees with inadequate time and energy to engage in safety behaviors (Trougakos et al., 2014). When an intrusion occurs, the social interaction can distract an employee from work. Once psychological disengagement begins, it becomes difficult for employees to fully reengage in safety-related work, even if they are still physically present at the workplace (Jett and George, 2003). Accordingly, employees feel that they have more work to complete than within the available time, reducing their likelihood of completing safety-related tasks. Following the reasoning that small talk disrupts work absorption, we posit small talk may indirectly hinder safety performance through lower work absorption.

*Hypothesis 6: small talk exerts negative effect on safety compliance (6a) and safety participation (6b) via work absorption.*

## Materials and methods

### Sample and procedure

An online survey was executed to gather data from 312 full-time employees in an Air Catering company in northern China. The company was selected due to its stringent aviation food safety requirements, where minor procedural deviations (e.g., temperature control lapses during meal assembly) can trigger cascading flight safety consequences. After acquiring the permission of managers, we described the survey for employees via Voov Meeting. We explained to all employees and guaranteed that the survey was voluntary, confidential, anonymous, and irrelevant to their performance evaluation. Subsequently, employees who consented to participate in the survey were directed to the WeChat Group. With a list of names from HR, codes were assigned to each participant. Measures of the different variables were randomized across participants to control for order bias (Dillman et al., 2014). To minimize potential common method biases and alleviate participants’ fatigue (Podsakoff et al., 2003, 2012), we adopted a three-wave method for the data collection, with each wave spaced 1 month. In time 1, we gathered demographic variables and small talk among coworkers. In time 2, psychological availability and work absorption were evaluated. In time 3, employees’ safety performance (safety compliance and safety participation) was measured.

The final sample comprised 136 valid questionnaires, with an overall response rate of 43.59%. Of the 136 participants, 58 (42.6%) were women, and 78 (57.4%) were men. There were 5 (3.7%) who were postgraduates, 52 (38.2%) who were undergraduates, and 79 (58.1%) who had graduated from junior college. They ranged in age from 18–27 years (11.8%), 28–37 years (39.7%), and 38 years and older (48.5%). 7.4% of participants had worked for less than 1 year, 11.8% for 2–5 years, 22.8% for 6–9 years and 58.1% for 10 years and more.

### Measures

To ensure the equivalence and appropriateness of the measures within Chinese context, we adopted translation and back-translation procedures to render all items from English into Chinese (Brislin, 1986). For all measures, we utilized a seven-point Likert-type scale ranging from 1 (completely disagree) to 7 (completely agree).

#### Small talk

A four-item scale (Cronbach’s *α* = 0.891) developed by Baer et al. (2020) was adopted to measure small talk. This scale includes the following sample item: “I had ‘water cooler’ talk with my coworkers.”

#### Safety performance

Safety performance was assessed using an 8-item scale from Neal and Griffin (2006), which had two dimensions of safety compliance (Cronbach’s *α* = 0.949) and safety participation (Cronbach’s *α* = 0.871), each consisting of 4 items. Example items were “I ensure the highest levels of safety when I carry out my job” and “I voluntarily carry out tasks or activities that help to improve workplace safety.”

#### Psychological availability

A six-item scale (Cronbach’s *α* = 0.915) developed by Byrne et al. (2016) was adopted to measure psychological availability. This scale includes the following sample item: “I am emotionally ready to deal with the demands of my work.”

#### Work absorption

A six-item scale (Cronbach’s *α* = 0.895) developed by Schaufeli et al. (2002) was adopted to measure work absorption. This scale includes the following sample item: “When I am working, I forget everything else around me.”

#### Control variables

In the present study, demographic variables such as gender, age, education, and working tenure were taken as control variables due to the potential influence on safety performance.

### Analytic strategy

First, confirmatory factor analyses (CFA) were executed using Mplus 8.0 (Muthén and Muthén, 2012) to assess the validity of the measures (Hooper et al., 2008). Second, path analysis and bootstrapping approach were conducted to test for the direct and indirect effects of small talk (Preacher and Hayes, 2008; Zhao et al., 2010).

## Results

### Confirmatory factor analysis

Confirmatory factor analysis (CFA) was conducted with Mplus 8.0 to evaluate the validity of five key constructs. First, a five-factor CFA model, including small talk, psychological availability, work absorption, safety compliance, and safety participation, was examined. As shown in Table 1, the results revealed a favorable fit for the theorized five-factor model (*χ^2^*(109) = 224.731, CFI = 0.936, TLI = 0.920, RMSEA = 0.088, SRMR = 0.053). Multiple comparisons with alternative models were made to confirm that the five-factor model was the optimal structure to apply. The results in Table 1 showed that the five-factor model fitted the data more effectively than any of the competing models, thereby supporting the validity of our specified measurement model.

**Table 1:** Results of confirmatory factor analysis.

Model	*χ^2^*	*df*	*χ^2^/df*	CFI	TLI	RMSEA	SRMR
Five-factor	224.731	109	2.062	0.936	0.920	0.088	0.053
Four-factor: SC + SP	305.085	113	2.700	0.893	0.871	0.112	0.068
Three-factor: SC + SP, PA + WA	494.914	116	4.267	0.789	0.753	0.155	0.095
Two-factor: SC + SP, ST + PA + WA	689.082	118	5.840	0.682	0.634	0.189	0.140
One-factor	987.132	119	8.295	0.517	0.448	0.232	0.168

ST, small talk; SC, safety compliance; SP, safety participation; PA, psychological availability; WA, work absorption.

### Descriptive analysis

Means, standard deviations, reliabilities, and zero-order correlations of variables are presented in Table 2. Small talk is positively related to psychological availability (*γ* = 0.430, *p* < 0.01), while negatively related to work absorption (*γ* = −0.482, *p* < 0.01). Psychological availability is positively associated with safety compliance (*γ* = 0.508, *p* < 0.01) and safety participation (*γ* = 0.623, *p* < 0.01). Work absorption is positively related to safety compliance (*γ* = 0.312, *p* < 0.01) and safety participation (*γ* = 0.434, *p* < 0.01).

**Table 2:** Means, standard deviation, reliabilities, and correlations.

Variables	1	2	3	5	6	7	8	9	10
Gender	1								
Age	0.055	1							
Education	0.093	−0.228^**^	1						
Tenure	−0.005	0.512^**^	0.074	1					
Small talk	−0.128	−0.136	0.063	0.067	***0.891***				
Safety compliance	−0.071	0.034	−0.146	−0.089	0.199^*^	***0.949***			
Safety participation	−0.233^**^	−0.004	−0.122	−0.081	0.240^**^	0.748^**^	***0.871***		
Psychological availability	−0.176^*^	−0.136	0.043	−0.056	0.430^**^	0.508^**^	0.623^**^	***0.915***	
Work absorption	−0.092	−0.092	0.007	−0.063	−0.482^**^	0.312^**^	0.434^**^	0.541^**^	***0.895***
Mean	0.426	4.015	1.456	3.316	3.594	4.574	4.350	4.279	3.733
*SD*	0.496	1.135	0.569	0.948	1.067	0.616	0.684	0.636	1.052

*N* = 136. Cronbach’s *α* values are shown along the diagonal in bold italics.

^*^p < 0.05, ^**^p < 0.01.

### Test of hypotheses

**Figure 2:**
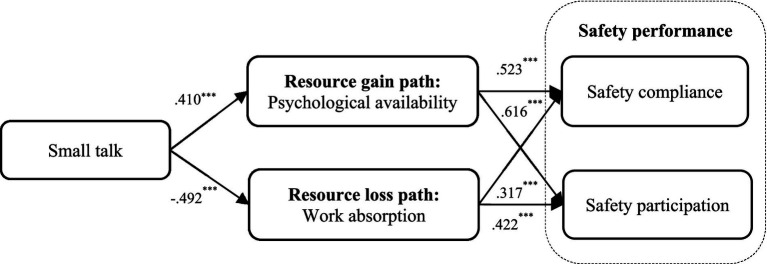
Results of path analysis.

Path analysis was utilized to verify hypotheses 1, 2, 4, and 5. As summarized in Figure 2, small talk positively predicted psychological availability (*β* = 0.410, *p* < 0.001), and negatively predicted work absorption (*β* = −0.492, *p* < 0.001) after including the controls. Thus, H1 and H4 were supported. The significantly positive effects of psychological availability on safety compliance (*β* = 0.523, *p* < 0.001) and safety participation (*β* = 0.616, *p* < 0.001) were confirmed. Hence, H2a and H2b were supported. Furthermore, the significantly positive effects of psychological availability on safety compliance (*β* = 0.317, *p* < 0.001) and safety participation (*β* = 0.422, *p* < 0.001) were verified. Accordingly, H5a and H5b were supported.

All remaining hypotheses were tested using the PROCESS macro in SPSS 25.0 (Hayes, 2013) with a 5,000-resample bootstrap method (Preacher et al., 2007). To verify hypothesis 3a and 3b, PROCESS model 6 was implemented. The result, as depicted in Table 3, demonstrated the significant indirect effect of psychological availability on the “small talk-safety compliance” relationship [E.S. = 0.117, 95% bias-corrected CI = (0.058, 0.200)] and “small talk-safety participation” relationship [E.S. = 0.146, 95% bias-corrected CI = (0.076, 0.239)]. Thus, H3a and H3b were supported.

**Table 3:** Results of mediating effects.

Structural path	Effect	SE	LLCI	ULCI
The mediating effect of psychological availability				
Small talk → psychological availability → safety compliance	0.117	0.037	0.058	0.200
Small talk → psychological availability → safety participation	0.146	0.041	0.076	0.239
The mediating effect of work absorption				
Small talk →work absorption → safety compliance	−0.017	0.032	−0.067	−0.060
Small talk → work absorption → safety participation	−0.050	0.035	−0.164	−0.040

Likewise, PROCESS model 6 was executed to verify hypothesis 6a and 6b. The result, as shown in Table 3, indicated the significant indirect effect of work absorption on the “small talk-safety compliance” relationship [E.S. = − 0.017, 95% bias-corrected CI = (−0.067, −0.060)] and “small talk-safety participation” relationship [E.S. = − 0.050, 95% bias-corrected CI = (−0.164, −0.040)]. Thus, H6a and H6b were supported.

## Discussion

Grounded in conservation of resources (COR) theory, this study advances a theoretical model to explore how small talk influences employees’ safety performance, empirically demonstrating its dual opposing effects through distinct mediating mechanisms: psychological availability and work absorption. Building on prior research, we provide robust evidence that small talk enhances safety performance by bolstering psychological availability to allocate cognitive and emotional resources to safety tasks, while simultaneously undermining it by reducing work absorption, the deep focus necessary for sustained task engagement. Our findings highlight the mediating processes through which informal social interaction shapes safety outcomes, offering a nuanced explanation of paradoxical role of small talk in the safety workplace. By linking small talk to both resource acquisition (via psychological availability) and resource depletion (via work absorption), this study validates core tenets of COR theory and illuminates how seemingly trivial interactions can produce both beneficial and detrimental effects on safety behavior. The dual pathway framework enriches our understanding of social dynamics in safety-critical environments, underscoring the need to balance relational benefits with cognitive demands to optimize workplace safety.

### Theoretical implications

In examining those hypotheses, the findings of our study make several critical theoretical implications for the research. First, we focus on small talk as a significant factor in improving employee safety performance represents a notable departure from traditional research approaches. Previous research has largely concentrated on elements such as leadership style, organizational culture, and job characteristics (Liu et al., 2022; Kalteh et al., 2021). In contrast, this study shifts the perspective to individual behavior, highlighting the importance of small talk. This shift in our study is not only a departure but also a complement to existing theories. While existing theories provide valuable insights into the various factors contributing to safety performance, they often overlook the crucial role of social interactions and communication (Guo et al., 2019; Lu and Kuo, 2016; Denner et al., 2025). Small talk, as a form of social interaction, bridges this gap by demonstrating its potential to directly influence employees’ attitudes and behaviors towards safety. Our study provides an additional dimension of understanding that enriches and extends the existing theoretical framework, allowing for a more comprehensive analysis of the complex relationships among individuals, their social environment, and safety performance.

Second, our study challenges the long-held view that small talk is universally negative or detrimental. Small talk is a complex social behavior that cannot be simply classified as unimportant or wasteful (Coupland, 2000; Methot et al., 2021). Our study reveals that small talk is, in fact, a multifaceted social behavior that defies simplistic categorization. On one hand, it can enhance social cohesion among employees and serve as a source of psychological resources. Friendly conversations during breaks can strengthen relationships, boost morale, and provide emotional support, all of which contribute to a more positive work environment conducive to safety. On the other hand, small talk can also disrupt employees’ work absorption. When it occurs during task-critical periods, it may distract employees from their work, reducing their focus on safety-related tasks and potentially decreasing safety awareness. By revealing the double-edged sword effect of small talk on employees’ safety performance, this study provides a more comprehensive and accurate understanding of this phenomenon. And, the dual perspective helps to overcome the one-sided view of small talk and encourages a more balanced consideration of its benefits and costs.

Third, our study validates and refines the mechanisms proposed by the COR theory. Our findings regarding the dual-edged effect of small talk on safety performance through resource loss and gain provide empirical evidence for the core principles of the COR theory. Specifically, our study illustrates that small talk can both deplete resources (reducing work absorption) and enhance resources (increasing psychological availability) (Hobfoll, 2011; Hobfoll et al., 2018). The dual-effect demonstration helps to further clarify and solidify the understanding of how resource dynamics operate within the COR theory. It allows for a more detailed examination of how different types of social interactions can trigger specific resource-related consequences and how these, in turn, affect performance outcomes.

### Practical implications

There are several important implications for managerial practices in our research. First, clear policies regarding small talk should be formulated in the workplace. These guidelines should delineate acceptable times and places for small talk to prevent it from disrupting work processes (Kim et al., 2017). For example, specific break areas or times can be designated, enabling employees to engage in casual conversations without impinging on their productivity or safety performance. Meanwhile, it is essential to minimize distractions in the workplace that can interfere with safety performance. It may involve ensuring that workstations are well-organized and clutter-free, and providing quiet zones for employees to focus on their tasks when required. For instance, installing soundproof partitions or providing noise-canceling headphones can assist in reducing noise levels and creating a more conducive work environment for employees.

Second, employees can take the initiative to use small talk to enhance psychological availability. They should actively seize the moments of small talk with colleagues as a way to improve their own psychological availability. Through interaction and sharing with colleagues, they can not only exchange valuable work-related experiences and insights but also obtain more information support and emotional resonance, thereby effectively strengthening their ability to manage and regulate their psychological states. The positive mindset and active interaction can greatly encourage employees to engage in safety behaviors with more enthusiasm and higher concentration (May et al., 2004).

Third, given that small talk may cause distraction, employees can adopt a series of effective strategies to quickly regain concentration, such as practicing deep breathing exercises, taking short breaks, or setting clear work goals (Kahn, 1990). At the same time, employees can also learn and master some professional skills to plan and allocate their work time more reasonably. Through such self-management and skill improvement, employees can further enhance work effectiveness and career development.

### Limitations and future research

The limitations in our study indicate several possible directions for future research. First, this study failed to identify specific partners with whom respondents engaged in small talk. The functions of small talk may vary depending on the nature of the relationship with the interaction partner (e.g., small talk with close friends vs. strangers) (Methot et al., 2021). Also, distinguishing between the initiator and receiver of small talk could clarify issues related to reverse causality, yet this was not done. Future research can adopt a dyad-level perspective to investigate partner-specific effects can be beneficial. It involves studying whether being the initiator or receiver of small talk has different effects on individuals, as well as its relationship with reverse causality.

Second, although the results of CFA (Table 1) revealed a good fit for the theorized five-factor model, common method variance could remain a potential concern since the data was collected from the same source. Several procedural remedies were executed to mitigate potential bias (Podsakoff et al., 2003): First, all participants were assured that the survey was voluntary, confidential, anonymous, and irrelevant to their performance evaluation to alleviate their evaluation apprehension or social desirability biases. Second, different instructions were employed to create psychological separation within the survey, making it unlikely for participants to perceive direct relationships among the variables. Nevertheless, we encourage future research to replicate the results based on different sources (i.e., employees, peers, and supervisors) through a multi-wave research design.

Third, the sample in this study was drawn from Chinese Air Catering companies. While enhancing the internal validity, it restricted the external validity of the study. To improve the external validity of this research, future studies should utilize samples from multiple companies, across various industries and regions for data collection to further test the findings of this study. By incorporating data from multiple countries with distinct cultural, economic, and social backgrounds, we can better account for the potential variations that might exist in different organizational settings.

Finally, this study centered on employees’ mental state but did not account for other potential moderating or mediating variables, such as personality traits, task complexity, and social support networks. Individual differences in personality traits (e.g., extraversion or neuroticism) can alter how employees interpret and engage in small talk, potentially amplifying or minimizing its effects. Similarly, task complexity may moderate the effects of small talk: in high-risk, cognitively demanding operations (e.g., chemical plant troubleshooting), even brief informal exchanges may exacerbate attentional depletion, whereas in routine tasks, such interactions can enhance vigilance through psychological resources. Furthermore, social support may mediate the effects of small talk by buffering resource loss or amplifying resource gains. Future research should examine these factors to explain the relationship between small talk and safety performance deeply.

## Data Availability

The raw data supporting the conclusions of this article will be made available by the authors, without undue reservation.
